# Screening of suppressors of *bax*-induced cell death identifies glycerophosphate oxidase-1 as a mediator of *debcl*-induced apoptosis in *Drosophila*

**DOI:** 10.18632/genesandcancer.68

**Published:** 2015-05

**Authors:** Jessie Colin, Julie Garibal, Amandine Clavier, Sébastien Szuplewski, Yanick Risler, Cécile Milet, Sébastien Gaumer, Isabelle Guénal, Bernard Mignotte

**Affiliations:** ^1^ Université Versailles St-Quentin, Laboratoire de Génétique et Biologie Cellulaire, Montigny-le-Bretonneux, France; ^2^ Ecole Pratique des Hautes Etudes, Laboratoire de Génétique Moléculaire et Physiologique, Montigny-le-Bretonneux, France; ^3^ Co-senior authors

**Keywords:** Apoptosis, Bax, Debcl, mutagenesis, Glycerophosphate oxidase

## Abstract

Members of the Bcl-2 family are key elements of the apoptotic machinery. In mammals, this multigenic family contains about twenty members, which either promote or inhibit apoptosis. We have previously shown that the mammalian pro-apoptotic Bcl-2 family member Bax is very efficient in inducing apoptosis in *Drosophila*, allowing the study of *bax*-induced cell death in a genetic animal model. We report here the results of the screening of a *P[UAS]*-element insertion library performed to identify gene products that modify the phenotypes induced by the expression of *bax* in *Drosophila melanogaster*. We isolated 17 putative modifiers involved in various function or process: the ubiquitin/proteasome pathway; cell growth, proliferation and death; pathfinding and cell adhesion; secretion and extracellular signaling; metabolism and oxidative stress. Most of these suppressors also inhibit *debcl*-induced phenotypes, suggesting that the activities of both proteins can be modulated in part by common signaling or metabolic pathways. Among these suppressors, *Glycerophosphate oxidase-1* is found to participate in *debcl*-induced apoptosis by increasing mitochondrial reactive oxygen species accumulation.

## INTRODUCTION

Major executioners of programmed cell death by apoptosis are relatively well conserved throughout evolution. However, the control of commitment to apoptosis exhibits some differences between organisms. During mammalian cells apoptosis, various key pro-apoptotic factors are released from the inter-membrane space of mitochondria (for review, see [1]). These factors include cytochrome c, Apoptosis Inducing Factor (AIF), Endonuclease G, Smac/DIABLO (Second mitochondria-derived activator of caspase/direct IAP-binding protein with low PI) and the serine protease Omi/HtrA2. Once released in the cytosol, cytochrome c binds to the WD40 domain of Apaf-1 and leads to the formation of a cytochrome c/Apaf-1/caspase-9 complex called “apoptosome”, in which caspase-9 (a cysteinyl aspartase) auto-activates to initiate a caspase activation cascade that will lead to cell death. Mitochondrial permeabilization is under the control of the Bcl-2 family of proteins. These proteins share one to four homology domains with Bcl-2 (named BH1-4) and exhibit very similar tertiary structures. However, while some of these proteins (such as Bcl-2) are anti-apoptotic, the others are pro-apoptotic and assigned to one of the following sub-classes: BH3-only proteins (such as Bid) and multi-domain proteins (such as Bax). During apoptosis, Bax translocates to the mitochondrial outer membrane, undergoes conformational changes, oligomerizes and finally allows the release of pro-apoptotic factors from the intermembrane space (review [2]). Anti-apoptotic proteins of the Bcl-2 family oppose this Bax-mediated mitochondrial release of apoptogenic factors while BH3-only proteins can activate Bax or inhibit anti-apoptotic proteins of the family.

In *C. elegans*, activation of the caspase CED-3 requires CED-4, the homologue of Apaf-1 but no cytochrome c. The Bcl-2 family protein CED-9 constitutively interacts with CED-4 and thereby prevents the activation CED-3. This repression of cell death is released upon binding of CED-9 to the BH3-only protein EGL-1, which induces a conformational change in CED-9 that results in the dissociation of the CED-4 dimer from CED-9. Released CED-4 dimers form tetramers, which facilitate auto-activation of CED-3 [3]. Although CED-9 appears bound to mitochondria, these organelles seem to play a minor role in apoptosis in *C. elegans*, contrarily to mammals [4].

The role of mitochondria in *Drosophila* programmed cell death remains more elusive [1, 5-7]. Cytochrome c does not seem crucial in the apoptosome activation [8, 9], which is mediated by the degradation of the caspase inhibitor DIAP1 by proteins of the Reaper/Hid/Grim (RHG) family. The apoptotic cascade appears somehow inverted between flies and worm/mammals. In these two last organisms, apoptosis regulators are relocated from mitochondria to the cytosol. Contrarily, *Drosophila* apoptosis regulators are concentrated at or around mitochondria during apoptosis. Indeed, targeting the RHG proteins Reaper (Rpr) and Grim to mitochondria seems to be required for their pro-apoptotic activity [10-12]. Furthermore, Hid possesses a mitochondrial targeting sequence and is required for Rpr recruitment to the mitochondrial membrane and for efficient induction of cell death *in vivo* [13].

The important role played in *Drosophila* by the mitochondria in apoptosis is also suggested by the mitochondrial subcellular localization of Buffy and Debcl, the only two members of the Bcl-2 family identified, so far, in this organism. Buffy was originally described as an anti-apoptotic Bcl-2 family member [14, 15], but it can also promote cell death [16-19]. Debcl (death executioner Bcl 2 homolog), is a multidomain death inducer [19-23] that can be inhibited by direct physical interaction with Buffy [14]. When overexpressed in mammalian cells, *debcl* induces both cytochrome c release from mitochondria and apoptosis. This protein interacts physically with anti-apoptotic members of the Bcl-2 family, such as Bcl-2 itself, in mammals. In *Drosophila*, Debcl is involved in the control of some developmental cell death processes as well as in irradiation-induced apoptosis [15, 18, 24].

We have previously shown in *Drosophila* that mammalian Bcl-2 inhibits developmental and irradiation-induced cell death [25] as well as *rpr*- and *bax*-induced mitochondrial membrane potential collapse [26]. Interestingly, we have shown that *bax*-induced cell death is mitigated by loss-of-function (LOF) mutations in genes encoding some components of the TOM complex which controls protein insertion in the outer mitochondrial membrane [5]. These results suggest that Bax mitochondrial location remains important for its activity in *Drosophila*. Therefore, flies provide a good animal model system to study Bax-induced cell death in a simple genetic background and look for new regulators of Bcl-2 family members.

Here, we report the results of the screening of *P[UAS]*-element insertion (UYi) library, performed in order to identify modifiers of *bax*-induced phenotypes in *Drosophila*. Among 1475 UYi lines screened, 17 putative modifiers were isolated, that include genes involved in various cellular functions. We also present a more detailed study of one of these modifiers, *UY1039*, and show that glycerophosphate oxidase-1 (Gpo-1) [EC 1.1.5.3] participates in *debcl*-induced apoptosis by increasing reactive oxygen species (ROS) production.

## RESULTS

### A modifier screen for suppressors of bax-induced phenotypes

We have previously shown that expression of the proapoptotic gene *bax* induced apoptosis in the developing eye or wing [25, 26]. Expression of *bax* under control of the wing specific *vg-GAL4* driver during development led to lethality and notches in the wing of the surviving escapers. As expected from the temperature sensitivity of the UAS-GAL4 expression system, lethality was more penetrant and wing phenotypes were more severe when flies were raised at 25°C than at 18°C. This adult wing phenotype was suppressed by *bcl-2* expression [25, 26] and by heterozygosity for LOF mutations in genes encoding Tom22 or Tom70 [5], indicating that the *vg>bax-*induced adult wing phenotype is sensitive and thus amenable to genetic screening.

**Figure 1:**
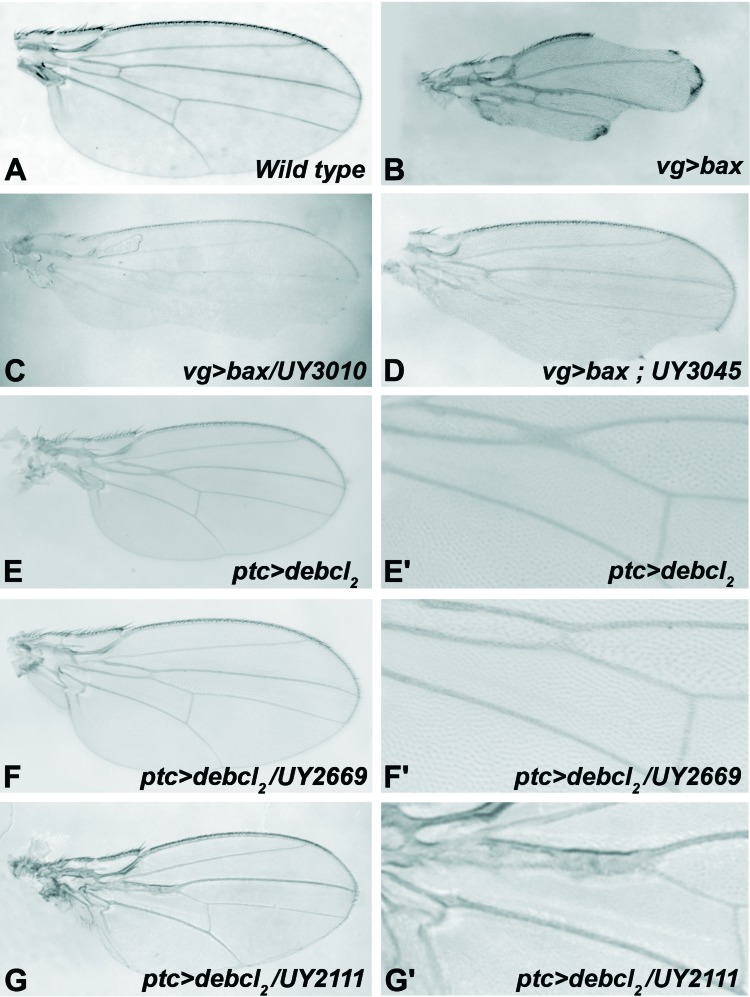
Examples of modified adult wing phenotypes **(A)** Wild-type wing. **(B-D)** Adult wings from *bax* expressing flies (at 18°C). **(B)** *vg*>*bax*, **(C)** *vg>bax/UY3010*, **(D)** *vg>bax* ; *UY3045.* **(E-G)** Adult wings from *debcl* expressing flies (at 25°C). **(E'-G')** are magnifications of **(E-G)**. **(E-E')** *ptc>debcl2* **(F-F')** *ptc*>*debcl2*/*UY2669*, **(G-G')** *ptc*>*debcl2*/*UY2111*.

To gain insight into the molecular mechanism of *bax*-induced apoptosis and with the aim of isolating regulators of this process, we designed a genetic screen for modifiers of Bax-mediated tissue loss in the wing. To ease the screening procedure, we used a strain recombined for *vg-GAL4* and *UAS-bax* transgenes. Animals heterozygous for *vg>bax* showed a strong and scorable notched wing phenotype (Figure 1B, compared to 1A), facilitating the selection of suppressors rather than enhancers.

A mutagenesis involving the transposition of a *P*-element containing *UAS* sequences, *P[Mae-UAS.6.11]* has been performed. Genes flanking the 5′ end of the transposon could therefore be transcriptionally regulated by the *UAS/GAL4* system and thus overexpressed. The *P*-element insertion, *per se*, could also generate LOF mutations. As part of a consortium of laboratories, we have produced *Drosophila* lines with random insertion sites of the *P*-element named hereafter UYi [27-30]. Thus, a collection of 1475 lines, *i.e*. 594 with the *P*-element inserted on the second chromosome, 775 on the third chromosome and 106 on the X chromosome was generated. Males carrying *UYi* were crossed with *vg>bax/CyOGFP* females and their progeny were screened for a rescue of both lethality (at 18°C and/or 25°C) and the notch phenotype (see Materials and Methods). Examples of wing phenotypes suppression are shown on Figure 1C and D. 56 *UYi* lines were selected, corresponding to 3,8% of the collection (56/1475).

Since the number of flies exhibiting a rescue of the wing phenotype was sometimes low, we tested the relevance of the suppression of the wing phenotype by a statistical approach. As previously described [26], expressivity and penetrance of the wing phenotype are variable in a population of flies of the same genotype. Flies expressing *bax* in the wing exhibit a distribution of phenotypes that can be classified into three categories: strong, intermediate and weak according to the number and size of notches observed along the wing margin. Therefore, we have used the statistical Wilcoxon test [31] to compare distributions of phenotypes between the two different types of progenies that express *bax* with or without the *UYi* suppressor. This test defines an α and a Ws value that respectively allow assessment of whether two distributions are significantly different or not, and which population is composed of stronger phenotypes. We defined the statistically significant limit as α<10^−3^. Using this stringent criterion, we identified 24 suppressors of *bax* acting both on fly survival and wing phenotype, among the 56 selected *UYi* lines, corresponding to 1.6% of the collection (24/1475).

Subsequently, to distinguish possible additive effects from more specific interactions, all selected UYi lines were crossed with *vg-GAL4*. Only wild type wing phenotypes were observed, ruling out an additive effect of *UYi* and *bax* transgenes, and thus revealing the specificity of the genetic interactions between *bax* and the 24 insertions.

To define if the wing phenotype suppression could be due to a gain of function (GOF) or a LOF, we identified the insertion point and orientation of each of the 24 insertions. PCR rescue experiments were performed and their products were sequenced. We compared the recovered sequence of the flanking genomic DNA to the *Drosophila* full-genome sequence database [32]. We were unable to obtain unambiguous flanking sequence information for 7 isolated *UYi* lines, *i.e*. *UY504*, *UY1220*, *UY1236*, *UY1649*, *UY2303*, *UY2650* and *UY2803*. Therefore, 17 putative suppressors of *bax*-induced apoptosis were identified. The results obtained during the screen of these bax-induced phenotypes are presented in table 1. Table 2 summarizes the genetic and molecular characterization of the selected insertions.

**Table 1:** Identified suppressors of bax-induced lethality and wing phenotypes The chromosomal location, the increase in survival rate at 18°C or 25°C as compared to *vg*>*bax* flies (NS: not significant) and the statistical result of the wing phenotype suppression are presented for each of the 17 identified suppressors.

Strain	Chromosome	Lethality suppression tests				Wing notches phenotype suppression(Wilcoxon test)	
		Increase in survival at 18°C		Increase in survival at 25°C			
**UY558**	II	67%	n=61	NS	n=62	α=7.9×10^−10^	n=125
**UY1039**	II	86%	n=151	118%	n=50	α= 8.2×10^−4^	n=173
**UY1116**	II	144%	n=110	NS	n=44	α=3.3×10^−4^	n=330
**UY1118**	II	70%	n=98	NS	n=46	α=5×10^−7^	n=125
**UY1131**	II	NS	n=88	215%	n=49	α=7.3×10^−5^	n=206
**UY1615**	II	52%	n=73	NS	n=55	α<10^−15^	n=229
**UY2056**	II	66%	n=64	133%	n=39	α=2×10^−6^	n=120
**UY2106**	III	NS	n=147	110%	n=79	α=2×10^−4^	n=144
**UY2111**	II	NS	n=82	148%	n=80	α<10^−15^	n=254
**UY2220**	III	NS	n=115	110%	n=38	α=2.9×10^−4^	n=125
**UY2510**	III	95%	n=112	77%	n=59	α= 6.3×10^−5^	n=418
**UY2564**	III	95%	n=56	NS	n=40	α=2.35×10^−5^	n=232
**UY2625**	III	108%	n=107	152%	n=57	α=1.85×10^−6^	n=497
**UY2669**	III	NS	n=170	174%	n=48	α=8.2×10^−5^	n=80
**UY3010**	II	56%	n=98	124%	n=59	α=5.1×10^−15^	n=243
**UY3045**	III	136%	n=73	90%	n=74	α=2.82×10^−5^	n=350
**UY4001**	X	275%	n=49	107%	n=38	α=1.3×10^−11^	n=255

**Table 2:** Insertion site of the *P[UAS]*-element in suppressors of *bax*-induced phenotypes Concerning UY2564 strain the *P* element insertion site could not be singled out by reverse PCR and two insertion sites remain possible.

Strain	Chromosome	Insertion site	Cytological location	Gene	Putative insertion effect
**UY558**	**II**	~1183988	21F2	*CG5126*	overexpression
**UY1039**	**II**	11749089	52C8	*Gpo-1*	loss of function
**UY1116**	**II**	5027391	25C1	*vkg*	overexpression
**UY1118**	**II**	~20779074	60E5	*Ance-5*	overexpression
**UY1131**	**II**	19157906	37C1-C6	*brat*	loss of function
**UY1615**	**II**	6421874	47A11-A13	*lola*	overexpression
**UY2056**	**II**	~1555073	22A3	*CG14351*	loss of function
**UY2106**	**III**	4495304	85A5	*CG8036*	overexpression
**UY2111**	**II**	~2108075	42A13	*bin3*	overexpression
**UY2220**	**III**	15612981	71E4	*comm3*	loss of function
**UY2510**	**III**	15721530	71F2	*comm*	loss of function
**UY2564**	**III**	10957668 /10960827	88E2	*CG6934/CG6912*	loss of function
**UY2625**	**III**	27811473	100E1	*heph/CG2003*	loss of function
**UY2669**	**III**	~3983390	64A2	*scrt*	overexpression
**UY3010**	**II**	5080803	46A1	*Uba1*	overexpression
**UY3045**	**III**	6957776	65D5	*sgl*	overexpression
**UY4001**	**X**	13716347	12C6	*clic*	loss of function

Candidate genes were regrouped according to the known or putative molecular function of their predicted products (Table 3). They are involved in cell growth, proliferation or death, pathfinding and cell adhesion, secretion and extracellular signaling, metabolism and oxidative stress, ubiquitin/proteasome pathway.

**Table 3:** Molecular function of *bax*-induced wing phenotype suppressors

Functional group	Strain	Gene	Molecular function / Biological processes
**Cell growth, proliferation or death**	UY1131	*brain tumor (brat)*	Translation repressor activity / negative regulation of cell proliferation
	UY4001	*clic*	Chloride channel activity / response to oxidative stress / apoptosis ?
	UY558	*CG5126*	Choline kinase / unknown
	UY2111	*bicoid-interacting protein 3 (bin3)*	S-adenosylmethionine-dependent methyltransferase activity / olfactory behavior
	UY1615	*lola*	RNA polymerase II transcription factor activity / axon guidance
**Pathfinding and cell adhesion**	UY2510	*commissureless (comm)*	Protein binding / axon guidance
	UY2220	*comm3*	Protein binding / salivary gland cell autophagic cell death
	UY2056	*hattifattener (hat)*	Receptor binding / axon guidance
	UY2669	*scratch (scrt)*	Transcription factor activity / dendrite morphogenesis
	UY1116	*viking (vkg)*	Type IV collagen / basal lamina component
**Secretion and extracellular signaling**	UY3045	*sugarless (sgl)*	UDP-glucose 6-dehydrogenase / proteoglycan biosynthetic process
	UY1118	*Ance-5*	Peptidyl dipeptidase activity / protein secretion
	UY2564	*CG6934/CG6912*	Growth factor receptor ?
	UY2625	*hephaestus (heph)/CG2003*	Poly-pyrimidine tract binding, mRNA binding / Notch signaling pathway
**Metabolism and oxidative stress**	UY1039	*Gpo-1*	Glycerophosphate oxidase-1/ carbohydrate metabolism
	UY2106	*CG8036*	Transketolase activity / pentose-phosphate shunt
**Ubiquitin proteasome pathway component**	UY3010	*Ubiquitin activating enzyme 1 (Uba1)*	Ubiquitin activating enzyme activity / proteasome pathway

### Study of the effects of bax suppressors on debcl-induced phenotypes

Bax seems to induce cell death in the eye thanks to its interaction with Debcl [24]. Moreover, only few data are reported concerning Debcl regulation and its partners. Therefore, we decided to test if the identified modifiers of *bax*-induced cell death could also genetically interact with *debcl*.

As for *bax*-induced cell death, the expression of *debcl* in wing imaginal discs during development induces apoptosis, which leads to a wing phenotype [14]. A very homogenous adult phenotype was obtained when expressing *debcl* along the antero-posterior frontier of wing discs, thanks to *ptc-GAL4* driver [33]. Under these conditions, *debcl* expression brought closer L3 and L4 veins in the proximal region of the wing, inducing a fusion of these veins in the region of the anterior cross vein (figure 1E and E'). These phenotypes were due to an excess of apoptosis [33].

Before testing genetic interactions, all selected *UYi* insertion were crossed with *ptc-GAL4* flies, to verify that their own overexpression did not give rise to any wing phenotype. All lines studied showed a wild type wing phenotype when tested with *ptc-GAL4*. An exception was *UY2669* that exhibited an absence of anterior cross vein but no alteration at the level of the antero-posterior frontier.

This secondary screen was performed on 15 insertions because two lines were lost (*UY1116* and *UY1039*). We found nine suppressors of *debcl*-induced phenotype (Table 4). An example is shown in Figure 1F-F' for *UY2669*. Four of Bax modifiers had no significant effect and two led to complex phenotypes of partial penetrance as shown for *UY2111* in Figure 1G-G'. Thus, most of *bax*-induced suppressors also suppressed *debcl*-induced apoptosis.

**Table 4:** Effect of Bax suppressors on *debcl*-induced phenotypes

	Strain	Gene	Insertion effect	Wilcoxon test
Suppressor	**UY558**	*CG5126*	overexpression	α=1.9×10^−9^ n=271
	**UY1118**	*Ance-5*	overexpression	α=9.5×10^−8^ n=270
	**UY1615**	*lola*	overexpression	α=5.1×10^−5^ n=242
	**UY2056**	*hat*	loss of function	α=1.3×10^−12^ n=242
	**UY2220**	*comm3*	loss of function	α=6.3×10^−6^ n=250
	**UY2510**	*comm*	loss of function	α=7.2×10^−5^ n=217
	**UY2564**	*CG6934/CG6912*	loss of function	α=6.6×10^−4^ n=229
	**UY2669**	*scratch*	overexpression	α<10^−15^ n=241
	**UY4001**	*clic*	loss of function	α<10^−15^ n=306
Complex phenotype	**UY2111**	*bin-3*	overexpression	na
	**UY2625**	*heph/CG2003*	loss of function	na
No significant effect	**UY1131**	*brat*	loss of function	α=0.027 n=216
	**UY2106**	*CG8036*	overexpression	α=0.027 n=206
	**UY3010**	*Uba1*	overexpression	α=0.002 n=247
	**UY3045**	*sugarless*	overexpression	α=0.512 n=243

### Glycerophosphate oxidase-1 participates in superoxide production during Debcl-induced apoptosis

**Figure 2:**
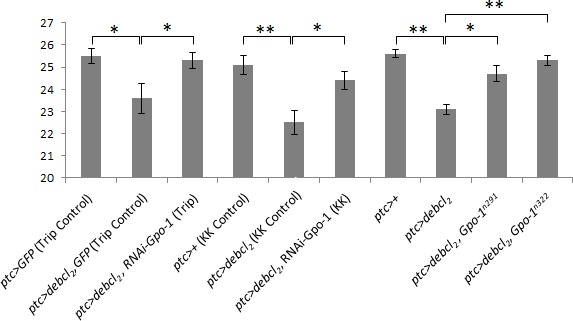
*Gpo-1* loss of function suppresses *debcl*-induced phenotypes Measure of relative distance between L3 and L4 veins in wings from *ptc>GFP* (Trip Control); *ptc>debcl2,GFP* (Trip Control); *ptc>debcl2,RNAi-Gpo1* (Trip); *ptc>+* (KK Control); *ptc>debcl2* (KK Control); *ptc>debcl2, RNAi-Gpo-1* (KK); *ptc>+*; *ptc>debcl2*; *ptc*>*debcl2,Gpo1291* and *ptc*>*debcl2,Gpo1n322* flies at 25°C. Error bars are the S.E.M. *: Student's t test α<0.05. **: Student's t test α<0.01.

**Figure 3:**
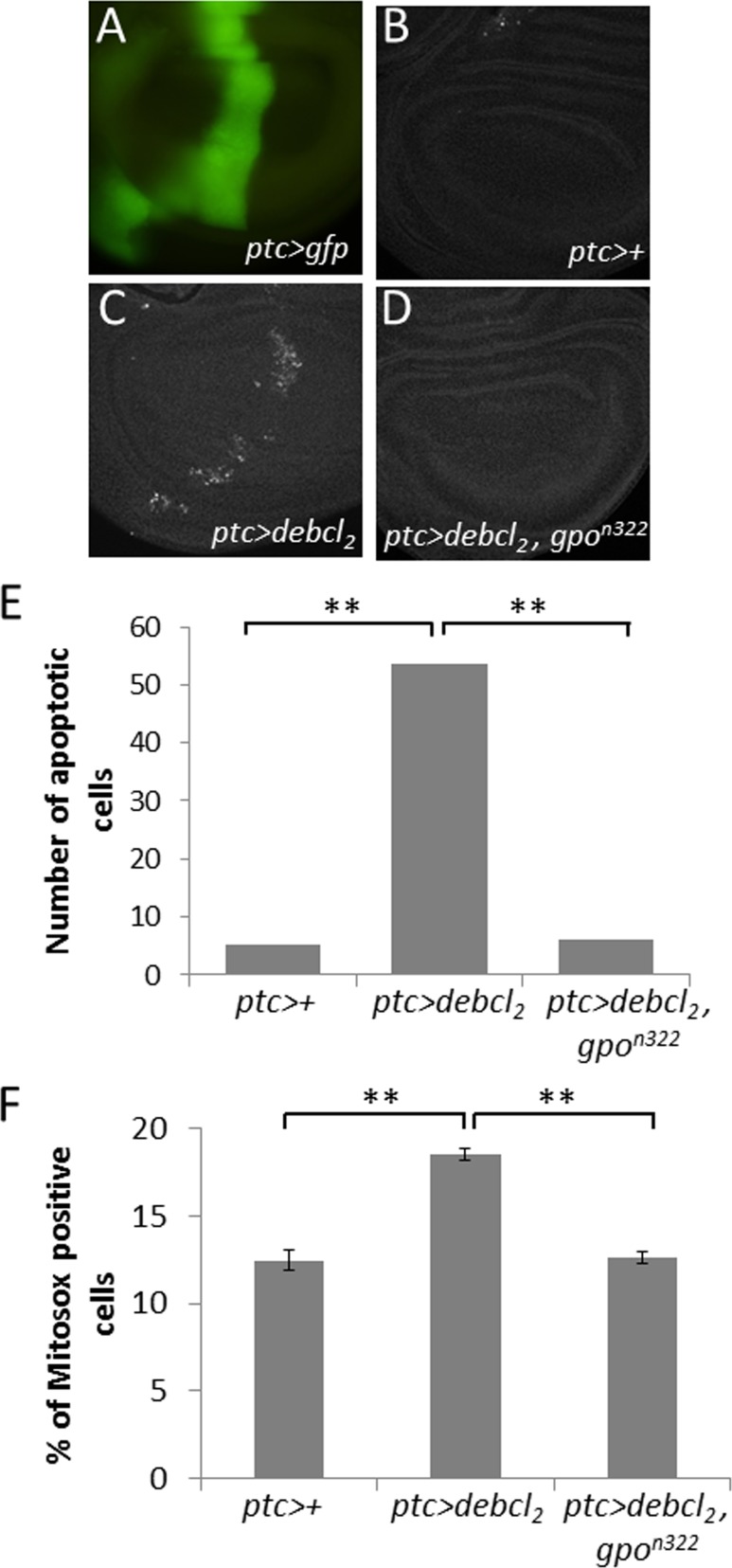
*Gpo-1* loss of function suppresses *debcl*-induced apoptosis by limiting mitochondrial ROS accumulation **(A)** *ptc* expression domain visualized by GFP fluorescence in a wing imaginal disc. **(B-D)** TUNEL staining of wing imaginal discs from *ptc>+*; *ptc>debcl2*; and *ptc*>*debcl2,Gpo1n322* wing imaginal discs. **(E)** Quantification of TUNEL positive cells of wing imaginal discs. **(F)** Quantification by flow cytometry of MitoSOX staining in larval wing imaginal discs. All these experiments were performed at 18°C. Error bars are the S.E.M. **: Student's t test α<0.01.

The line *UY1039* being lost we assumed that the suppressor effect of *UY1039* was due to a LOF mutation in the *Glycerophosphate oxidase-1* gene. Given that this gene is involved in the mitochondrial metabolism, and that numerous Bcl-2 family members act at the mitochondrial level, Gpo-1 seemed of high interest and we decided to focus on its study. To confirm our hypothesis, we tested whether RNAi against *Gpo-1* or heterozygosity for a *Gpo-1* hypomorph (*Gpo-1^291^*) or an amorph (*Gpo-1^n322^)* allele, could suppress *Debcl* overexpression-induced wing phenotype. Both RNAi and both *Gpo-1* LOF heterozygous alleles suppressed *debcl*-induced phenotypes but the most complete and fully penetrant suppression was observed in flies heterozygous for the *Gpo-1^n322^* mutation (Figure 2). Therefore, we decided to assess the apoptosis level in wing imaginal discs of flies heterozygous for *Gpo-1^n322^*. The number of TUNEL positive cells in wing discs overexpressing *debcl* was dramatically reduced by *Gpo-1^n322^* heterozygosity when compared to discs that are not mutated in *Gpo-1* (Figure 3A-E), thus confirming that a reduction of *Gpo-1* dosage suppresses *debcl*-induced apoptosis.

Gpo-1 [EC 1.1.5.3], also known as glycerol-3-phosphate dehydrogenase, is encoded by a nuclear gene and located at the outer surface of the inner mitochondrial membrane. It catalyzes the reaction:

sn-glycerol 3-phosphate + coenzyme Q10 -> glycerone phosphate + reduced coenzyme Q10

Along with the cytosolic NAD-linked glycerol phosphate dehydrogenase (GPDH), Gpo-1 forms the glycerol phosphate shuttle that catalyzes the interconversion of glycerol phosphate and dihydroxyacetone phosphate to oxidize cytosolic NADH by transferring reducing equivalents from the cytosol to mitochondria. Cytosolic GPDH has been shown to protect mammalian CHO cells against ROS-induced apoptosis [34]. On the opposite, mitochondrial Gpo-1 has been involved in superoxide production in various species (review: [35]) including *Drosophila* [36], which is in agreement with a protective role of a dosage reduction of this gene. Furthermore, we have observed that *debcl* overexpression can induce ROS accumulation thanks to a mitochondrial superoxyde indicator, *i.e.* MitoSOX. Indeed, *debcl* overexpression increases the proportion of MitoSOX positive cells in the wing imaginal discs compared to the *ptc-gal4/+* control (Figure 3F). Thus, we decided to test whether heterozygosity for the *Gpo-1* null mutant could decrease ROS levels produced by *debcl* overexpression. A significant decrease of MitoSOX positive cells was observed when *debcl* was overexpressed in a *Gpo1^n322^* heterozygous background when compared to Gpo-1^+/+^ background (Figure 3F). This result indicates that *Gpo-1* participates in *debcl*-induced apoptosis by increasing ROS production.

## DISCUSSION

This screen provides us with 17 suppressors of phenotypes induced by the expression of *bax* under control of the wing specific *vg-GAL4* driver (lethality and wing notches). The possibility that these suppressors affect GAL4 synthesis or that the selected insertions titrate the GAL4 transcription factor is unlikely, since our number of suppressors is limited (1.6% of the collection). Moreover, we isolated *UYi* insertions, which were not identified in other screens performed using the same collection and the UAS/Gal4 system [28, 30]. Finally, we have recently reported the specificity of one of the suppressors, *UY3010*, which corresponds to a gain-of-function of the Ubiquitin activating enzyme-encoding gene *Uba1*. Indeed, *Uba1* overexpression allows the degradation of Bax and Debcl, thanks to the activation of the ubiquitin/proteasome pathway [33]. We also showed in this study that Debcl is targeted to the proteasome by the E3 ubiquitin ligase Slimb, the β-TrCP homologue [33].

We found that 9 of the *bax*-modifiers also behaved as suppressors of *debcl*-induced wing phenotype while 4 showed no significant effect on this phenotype. Three hypotheses could explain this discrepancy. One possibility is that these *bax* modifiers are context artifacts and do not represent *bona fide* Bax interactors. The second possible explanation involves the difference in the driver used in each assay (*vg-GAL versus ptc-GAL*). Indeed, *UY3010* did not significantly suppress *debcl*-induced apoptosis while another *Uba1* overexpression mutant (Uba1^EP2375^) did [33]. Third, although Bax and Debcl, share similarities in their mode of action and regulation, some signaling pathways could be specific of *bax*-induced apoptosis. Indeed, a LOF of *brat* mitigates neither *debcl*- (this paper) nor *hid*- or *Sca3*-induced cell death [37].

The *brat* gene belongs to a group of suppressors, which is implicated in cell growth, proliferation or death. Mutations in this type of genes could compensate cell loss due to ectopic apoptosis induction. Results observed for this group of modifiers can generally be easily interpreted with the literature data. *UY1131* corresponds to an insertion in the *brat* (for *brain tumor*) gene that could allow the expression of a truncated form of the protein. To check whether this insertion leads to a LOF or a GOF of *brat*, we tested the effect of the characterized LOF allele *brat^k0602^* on *bax*-induced phenotypes. This mutation strongly suppressed (α=7.3×10^−7^) the wing phenotype showing that *UY1131* is a LOF of *brat* (data not shown). Brat belongs to the NHL family of proteins, represses translation of specific mRNAs 38] and is a negative regulator of cell growth [39-41]. The suppression of *bax*-induced phenotypes by a LOF of *brat* could suggest that this gene also regulates cell death, which seems unlikely according to its inability to suppress other cell death pathways [37]. Alternatively *brat* could regulate somehow compensatory proliferation in this system.

Some candidate suppressors encode proteins involved in secretion or components of the extra-cellular matrix. The effect of these genes could rely on cell signaling. Change in levels of secreted proteins could modify cell-extracellular matrix interactions and thus affect viability *via* processes similar to anoikis.

Several suppressors are implicated in pathfinding (*comm*, *comm3*, *hat*, *scratch* and *lola*). Two hypotheses can be formulated. Either neurons are of particular importance in *bax*-induced phenotypes or a more general role of these proteins in signaling is responsible for these suppressions. If the neuronal death could explain the decreased survival of *bax* expressing flies, it could hardly explain the wing phenotypes. Therefore, these suppressor genes may have a more general role in signaling and in particular in cell death regulation. For example, *UY2669* corresponds to a GOF mutant of *scratch* (*scrt*). This gene is a *Drosophila* homologue of *C. elegans ces-1*, which encodes a snail family zinc finger protein involved in controlling programmed death of specific neurons [42]. Interestingly, a mammalian homologue of *scratch*, named *Slug*, is involved in a survival pathway that protects hematopoietic progenitors from apoptosis after DNA damage [43]. *Slug* also antagonizes p53-mediated apoptosis by repressing the *bcl-2*-family pro-apoptotic gene *puma* [44]. More recently, a regulatory loop linking p53/Puma with Scratch has been described in the vertebrate nervous system, not only controlling cell death in response to damage but also during normal embryonic development [45].

Another possibility is that these modifiers could affect some extracellular survival and/or death factors. For example, *sugarless*, which was found twice in the screen, has been shown to interact with several survival pathways such as Wingless, EGF and FGF pathways that can play a role in defining shape and size of tissues and organs. This result can be paralleled with the suppressive effect of mutations in *hephaestus* and *lola*, both of which interact with the Notch/Delta signaling. Notably, *lola,* a gene encoding a Polycomb group epigenetic silencer, has been shown to be required for programmed cell death in the *Drosophila* ovary [46]. Lola has also been identified for its role in normal phagocytosis of bacteria in *Drosophila* S2 cells and as a component of the *Drosophila* Imd pathway that is key to immunity [47]. In contrast, Lola is required for axon growth and guidance in the *Drosophila* embryo [48]. This indicates that *lola* could play a role in cell adhesion and motility. Accordingly, when coupled with overexpression of *Delta*, misregulation of *pipsqueak* and *lola* induces the formation of metastatic tumors associated with a downregulation of the *Rbf* (*Retinoblastoma-family*) gene [49].

Other identified genes are involved in carbohydrate metabolism (*Gpo-1* and *CG8036* described as a transketolase). This result is in agreement with the evidence that Bcl-2 family proteins, in addition to their well characterized function in cell death, also play roles in metabolic processes in particular at the level of energetic metabolism (reviewed in [50]). In particular, Bcl-2 regulates mitochondrial respiration and the level of different ROS through a control of cytochrome c oxidase activity [51]. Study of heterologous *bax* expression in yeast has provided clues on Bax function in relation to ROS (reviewed in [52, 53]) and yeast LOF mutants of genes involved in oxidative phosphorylation show increased sensitivity to Bax cytotoxicity [54]. In agreement, Bcl-xL complements *Saccharomyces cerevisiae* genes that facilitate the switch from glycolytic to oxidative metabolism [55]. Furthermore, both the anti-apoptotic effect of LOF mutations in *Gpo-1* and the GOF in transketolase genes can be related to a protective effect against oxidative stress. This result suggests that the cell death process induced by Bax involves, at least in part, the modulation of different ROS levels.

Indeed, we report here that the suppressor effect of a null allele of *Gpo-1* is associated with a decreased ability of Debcl to induce ROS production. This result is in agreement with the observation that 70% of the total cellular H*^2^*O*^2^* production was estimated to stem from Gpo-1 in isolated *Drosophila* mitochondria [36]. This enzyme has also been implicated in ROS production in mammalian brown adipose tissue mitochondria when glycerol-3-phosphate was used as the respiratory substrate [56] and, more recently, in prostate cancer cells [57]. In this latter case, ROS production seems to be beneficial to cancer cells, whereas we show here that it favors cell death in *Drosophila* wing disc cells. This apparent contradiction could be related to the abnormal ROS production occurring during the oncogenic transformation and the shift to a glycolytic metabolism.

In conclusion, this study shows that Gpo-1 contributes to *debcl*-induced apoptosis by increasing reactive oxygen species (ROS) production and provides a substantial resource that will aid our efforts to understand the regulation of pro-apoptotic members of the Bcl-2 family proteins.

## MATERIALS AND METHODS

### Fly stocks

All strains were raised on standard culture medium at 25°C or 18°C. Generation of UYi lines was performed by standard *P* mobilization [58] of a *P[Mae-UAS.6.11]* [27]. The driver strains used in this study are *vestigial-GAL4* (*vg-GAL4*) [25] and *patched-GAL4* (*ptc-GAL4*) given by L. Théodore. The strain carrying *UAS-bax* (from mouse origin) has been generated in our laboratory and was previously described [25]. The strain carrying two insertions of *UAS-debcl-HA* (one on the second and one on the third chromosome) was given by H. Richardson [20] and recombined with the *ptc-GAL4* driver to generate the *pct>debcl^2^* strain. Either *y,w^c^* or *w^1118^* Canton S was used as the control strain according to the genetic background of the tested lines. The *Uba1^EP2375^* mutant strain was obtained from the Szeged Drosophila stock center. *Gpo-1^n322^* [59], *Gpo-1^291^* and *UAS-RNAi-Gpo-1* Trip #55319 strains were obtained from the Bloomington Drosophila Stock Center. The *UAS-RNAi-Gpo-1* VDRC KK #110608 strain was obtained from Vienna Drosophila RNAi Center.

### Screen for suppressors of lethality

UYi lines including a *P*-element insertion on the X, second or third chromosome were tested. Concerning the screening of UYi lines carrying a *P*-element on the second chromosome, the survival rate in the progeny was evaluated as follows. Each cross was performed with 7 virgin females and 3 males. [*vg>bax/CyOGFP*] virgin female flies were mated to [*UYi*/*UYi*] males when homozygous males were viable or, alternatively, to heterozygous [*UYi*/*CyO*] males. In the first case, the survival rate corresponds to ([*vg>bax*/*UYi*] / [*UYi*/*CyOGFP*]) while in the case of heterozygous mutant strains it corresponds to (([*vg>bax/CyO*] + [*vg>bax*/*UYi*]) / [*UYi*/*CyOGFP*]).

For UYi lines carrying the *P*-element on the third chromosome, virgin female flies [*vg>bax*/*CyOGFP* ; *+/+*] were mated to viable [*+/+* ; *UYi*/*UYi*] males or, alternatively, to [*+/+* ; *UYi*/*TM3*] males. In the first case, the survival ratio corresponds to ([*vg>bax/+ ; UYi/+*] / [*CyOGFP/+ ; UYi/+*]), while in the second case it was (([*vg*>*bax*/+ ; *UYi* /+] + [*vg*>*bax*/+ ; *TM3*/+]) / ([*CyOGFP*/+ ; *UYi* /+] + [*CyOGFP*/+ ; *TM3*/+])).

UYi lines located on the *X* chromosome were screened by crossing virgin [*UYi*/*UYi ; +/+*] or [*UYi/FM0 ; +/+*] female flies with [*+/Y ; vg>bax/CyOGFP*] males. If the mutant line was homozygous, the survival ratio was (([*UYi/+ ; vg>bax/+*]) / [*UYi/+ ; CyOGFP/+*]). If the mutant line was balanced with *FM0*, the ratio was (([*UYi/+ ; vg>bax/+*] + [*FM0 /+ ; vg>bax/+*]) / ([*UYi/+ ; CyOGFP/+*] + [*FM0/+ ; CyOGFP/+*]).

For *UYi* insertions located on autosomes, only crosses giving rise to a progeny of at least 25 individuals including at least two [*vg>bax*/*UYi*] were taken into account. For mutations of the X chromosome, only crosses giving rise to at least 25 individuals including at least 20 [*UYi*/+ ; *CyOGFP* /+] flies were taken into account.

To study phenotypes and survival, crosses were performed at 18°C and after five days they were either kept at 18°C or switched to 25°C. *UYi* mutations were considered as suppressors of lethality if the survival rate was higher than the observed average survival rate plus one standard deviation, either at 18°C or 25°C.. The average survival rate and standard deviation were independently determined for each of the three chromosomes bearing the UYi transgene to take into account the genetic background impact.

### Classification of the wing phenotypes and the Wilcoxon test

All the lineages were analyzed in parallel with a control progeny and by a blind observer. Flies were classified according to their wing phenotype (strong, intermediate or weak) as previously described [26]. For flies showing two different wing phenotypes, the strongest phenotype of both wings was used for classification. In our screen, we have analyzed the distribution of graded phenotypes based on their expressivity. The Wilcoxon test was used to compare the distributions of the phenotypes between two lineages A and B [31]. The sign of the Ws*^A-B^* value determines whether the distribution A is stronger than B (Ws<0) or whether the distribution B is stronger than A (Ws>0). We considered the difference between A and B significant when α*^A-B^*<10^−3^.

### Molecular characterization of UYi lines

To characterize the genes identified by screening for *bax*-induced phenotype modifiers, the DNA flanking the *P[Mae-UAS.6.11]* element was isolated by inverse polymerase chain reaction (PCR), essentially according to the Berkeley *Drosophila* Genome Project (BDGP) protocol (http://www.fruitfly.org/about/methods/inverse.pcr.html) and sequenced. The following primers were used in this study:

5′-GCAGTTGATTTACTTGGTTGCTGG-3′,

5′-GGTAAGCTTCGGCTATCGAC-3′,

5′-GCTTTCGCTTAGCGACGTGT-3′,

5′-GCTTTCGCTTAGCGACGTG-3′,

5′-GTATACTTCGGTAAGCTTCG-3′,

5′-CTCTCAACAAGCAAACGTGC-3′,

5′-ACACAACCTTTCCTCTCAACAA-3′,

5′-GAATTGAATTGTCGCTCCGT-3′,

5′-ATTGATTCACTTTAACTTGCAC-3′.

Sequencing was performed by Genecust (Genopole, Evry, France). Sequences were submitted to BLAST search in the BDGP database to identify nearby genes.

### Quantification of ptc-Gal4>(UAS-debcl)2-induced phenotype in the wing

To test the implication of *Gpo-1* in *debcl*-induced apoptosis, the severity of the wing tissue loss induced by *UAS-debcl* overexpression led by *ptc-Gal4* driver was measured in different genetic backgrounds. We first verified that the *Gpo1^n322^* LOF mutation did not induce any wing phenotype by itself. Then, *ptc>debcl^2^*, females were crossed with wild-type males or males bearing a LOF mutation for *Gpo-1.* For each progeny, the distance between veins L3 and L4 was measured perpendicularly to the sixth sensilla of the dorsal row of the anterior wing margin and plotted against the distance between the extremity of veins 4 and 5. Student's t-tests were then performed.

## TUNEL staining

As previously described [60, 61], third instar larvae were dissected in PBS pH 7.6, fixed in PBS/formaldehyde 3.7%, washed three times for 10min in PBT (1X PBS, 0.5% Triton). Discs were then dissected and TUNEL staining was performed according to manufacturer's instructions (ApopTag Red in situ apoptosis detection kit, Millipore, Temecula, CA, USA). Discs were mounted in CitifluorTM (Biovalley, Marne-La-Vallée, France) and observed with a Leica SPE upright confocal microscope (Leica, Wetzlar, Germany). White patches in the *ptc* expression domain were counted for at least 30 wing imaginal discs per genotype. Student's t-tests were then performed.

### Mitochondrial superoxide measurement

MitoSOX (Molecular Probes, Thermo Fisher Scientific) was used to measure the mitochondrial production of superoxide as described in [62]. Briefly, twenty wing imaginal discs were dissected in Schneider's *Drosophila* medium (Fisher Bioblock scientific, Illkirch Graffenstaden, France), then cells were trypsinized. 5 μM MitoSOX were added to the cells placed at 37°C. Red fluorescence was then measured by flow cytometry in 2000 events per experimental condition. Flow cytometric measurement was performed using a BD LSRFortessa (Becton Dickinson, Franklin Lakes, USA). Fluorescence was induced by the Yellow-Green Laser (561 nm). Red fluorescence was collected with a PE detector (emission: 578 nm).
